# Prolonged survival after multimodal therapy for pleural mesothelioma: Don't give up the follow-up. A case report

**DOI:** 10.1016/j.amsu.2020.11.034

**Published:** 2020-11-11

**Authors:** Julien Guinde, Bertrand Chollet, Sophie Laroumagne, Hervé Dutau, Philippe Astoul

**Affiliations:** a Department of Thoracic Oncology, Pleural Diseases, and Interventional Pulmonology, Hôpital Nord, Aix-Marseille University, Marseille, France; b Aix-Marseille University, Marseille, France

**Keywords:** Mesothelioma, Trimodal therapy, Long survival, Recurrence

## Abstract

Malignant Pleural mesothelioma (MPM) is a rare disease which is associated with a poor prognosis. Front line chemotherapy represents the cornerstone in the management of MPM, and the place of radical surgery is controversial and reserve in early-stage disease. However prolonged survival (more than 24 months) can be observed in rare cases and only in the context of multimodal treatment including surgical management. We report the case of a patient suffering from an epithelial MPM with a 14-years progression-free survival after trimodal treatment including extrapleural pneumonectomy followed by chemotherapy and radiotherapy. This case illustrates that despite being an aggressive disease, multimodal management including radical surgery may allow a prolonged response in MPM but requires a whole-life surveillance.

## Highlights

• Malignant pleural mesothelioma (MPM) is a rare disease.
• Prognosis of MPM is very poor despite new therapeutic agents.
• The place of radical surgery still remains controversial.
• Prolonged survival can be obtained by multimodal therapy including radical surgery.
• Complete remission after treatment requires a whole-life surveillance.

## 1 Introduction

Malignant mesothelioma is a rare disease of the pleura with a poor prognosis despite various treatments. Among them radical surgery remains controversial even in the setting of multimodal approach. However, the patients with prolonged survival are those managed with multimodal approach including extrapleural pneumonectomy (EPP) or pleurectomy/decortication (P/D). Usually these patients are macroscopically considered with complete tumoral resection (R0) and the challenge is the design of post therapeutic follow-up in terms of frequency and duration. Here we present, after his informed consent and according to SCARE criteria [1], the case of a patient who underwent EPP follow by adjuvant chemotherapy and radiotherapy with an extended survival and a disease recurrence 14 years after the completion of trimodal treatment.

## 2 Presentation of case

**Fig. 1:**
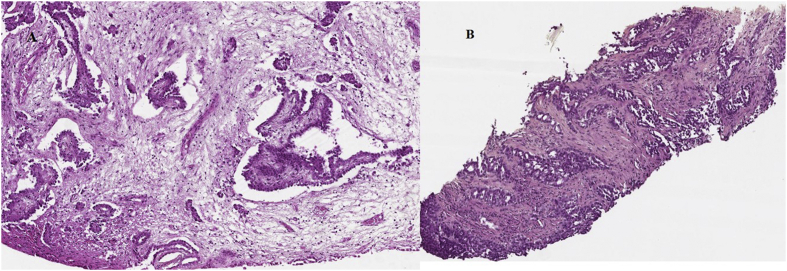
Histology at the initial diagnosis (A) and at the time of the recurrence (B). A. MPM epithelial sub-type consisting of papillary () and tubular structures with myxoid stromal feature (*). B. Epithelial Malignant Mesothelioma recurrence in a more acinar pattern (arrow) (HES).

**Fig. 2:**
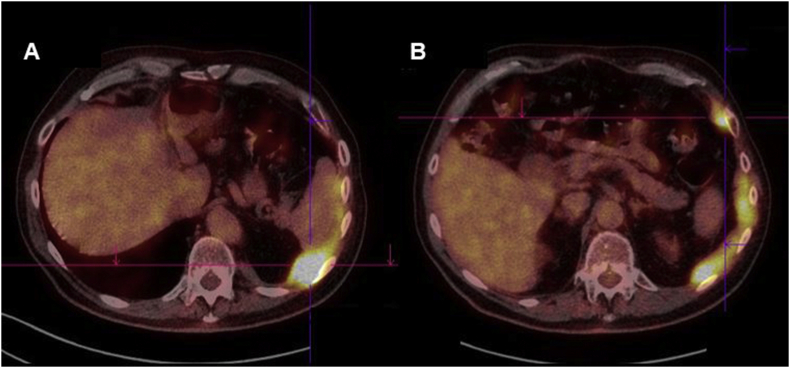
^18^F-fluorodeoxyglucose (FDG)- PET with computed tomography show a metabolic nodular localized (A) and diffuse (B) thickening of the left costo-diaphragmatic junction.

A 68-year-old man with a past-history of asbestos exposure was diagnosed in august 2004 with a malignant mesothelioma by pleuroscopy carried out for a left pleural effusion. Histology showed mesothelioma of epithelial type consisting of papillary and tubular structures (Fig. 1A). The tumor expressed the mesothelial markers of Calretinin, CK 5/6 and WT1. Confirmation of the diagnosis was obtained from the MESOPATH National Reference Center [2]. The patient was in excellent shape without major co-morbidity. Thoraco-abdominal CT and brain MRI showed no metastasis. Pulmonary function tests and cardio-vascular work-up were normal. The IMIG clinical stage was considered II [3]. Our MDT found the patient fitting for a multimodal treatment combining Extra Pleural Pneumonectomy (EPP) followed by chemotherapy and radiotherapy. After patient's informed consent the patient underwent an EPP in October 2004 which confirmed the stage of the disease (T2N0). The resection was considered R0. Four weeks after the surgery, a first cycle of chemotherapy, combining raltitrexed (3 mg/m^2^) and oxaliplatin (130 mg/m^2^), was administered followed three weeks after by radiotherapy with 65 Gy of the involved hemithorax for 6 weeks. Three other cycles of chemotherapy was administered starting four weeks after the completion of radiotherapy with mild hematologic toxicity and no alopecia [4]. The patient's follow-up began in June 2015 with a quarterly clinical examination and thoracic CT-scan assessment during the first year, same biannual assessment the next two years and after annual evaluation. At the start of 2018 a ^18^F-fluorodeoxyglucose (FDG) PET showed a metabolic nodular thickening of the left costo-diaphragmatic junction (Fig. 2). Histology obtained by percutaneous needle biopsy was consistent with a malignant mesothelioma of epithelial sub-type (Fig. 1B). The patient, 81 years-old, had an ECOG Performance Status 0 and no other co-morbidity. Our MDT indicated chemotherapy with carboplatin and pemetrexed every 21 days for 6 cycles with a stabilization of the disease for 10 months before a new progression. Palliative care was decided.

## 3 Discussion

The standard of care since the early 2000s is a platinum-based chemotherapy regimen [4,5]. A recent study showed that the addition of bevacizumab to pemetrexed plus cisplatin significantly improved overall survival in malignant pleural mesothelioma at the cost of expected manageable toxic effects [6]. However, in this setting, prolonged progression-free survival are very scarce. Surgery for MPM, EPP or extended P/D, is usually indicated in multimodal approaches and ideally in clinical trial settings despite the outcomes of MARS trial providing no evidence of benefit, for survival or quality of life, from EPP within trimodal therapy over chemotherapy alone [7]. Reports for local recurrence rates after EPP and RT (with or without neoadjuvant or adjuvant chemotherapy) range from 9% to 41%. The common site of recurrence after extrapleural pneumonectomy and planned multimodality therapy remains the ipsilateral hemithorax (including mediastinum), and true distant failure (other than the abdomen or contralateral hemithorax) remains unusual. These results impact the modality of the surgery arguing that extended P/D which is a less radical procedure, although with higher local recurrences, might be a best surgical option. In maintaining a good PS, the patients could receive post recurrence treatment with less complications which is a significant prognostic factor for post recurrence survival, yielding long-term outcomes after recurrence. In an effort to decrease the local recurrence rate after surgery, radiation therapy (RT) has been considered after surgery. However if hemithoracic RT may be offered to patients after EPP, it is a challenge after extended P/D, even the last technological developments in this field such as IMRT which is associated with low rates of locoregional recurrence but life-threatening lung toxicity for some patients [8]. In the current case, a trimodality approach was decided. At that time (2004) a preoperative work-up combining CT scan of the chest and upper abdomen, laboratory blood tests and pulmonary function tests was done according to the IMIG [3]. Interestingly the clinical classification was in accordance to the pathological classification. A correct staging of MPM is a major step in determining which treatment modalities would be the most appropriate when a surgery is discussed. For mesothelioma patients operated on, there are no validated recommendations regarding the follow-up. Our MDT decided a close surveillance the first 3 years mainly based, beside clinical examination, on CT-scan followed by annual CT-scan after taking into account that it is likely that many recurrences are asymptomatic and detected by imaging alone. The British Thoracic Society guidelines recommend patients should be offered 3 to 4 monthly follow-up appointments with an oncologist and/or respiratory physician according to their treatment plan [9].

## 4 Conclusion

Our case illustrates that: first, multimodal approach including radical surgery for the treatment of MPM can lead to a complete and prolonged response. Radical surgery must remain an option for operable patient in particular at the era of new therapeutic agents (biotherapy, immunotherapy). Second, a recurrence can happened after a prolonged period leading to the necessity of whole-life surveillance. To our knowledge, it is the first case concerning a 15-year prolonged progression-free survival in patient suffering from a stage II malignant pleural mesothelioma.

## Funding

None.

## Consent

Written informed consent was obtained from the patient for publication of this case report and accompanying images. A copy of the written consent is available for review by the Editor-in-Chief of this journal on request.

## Ethical approval

Not applicable.

## Author contribution

JG and PA collected the clinical data and wrote the paper.

PA followed the patient for 14 years.

BC, SL, HD approved the draft and contribute to this report.

## Provenance and peer review

Not commissioned, externally peer-reviewed.

## Guarantor

Philippe ASTOUL is the guarantor of this work.

## Declaration of competing interest

None.
